# Relationship Between Depression and the Use of Mobile Technologies and Social Media Among Adolescents: Umbrella Review

**DOI:** 10.2196/16388

**Published:** 2020-08-26

**Authors:** Jorge Arias-de la Torre, Elisa Puigdomenech, Xavier García, Jose M Valderas, Francisco Jose Eiroa-Orosa, Tania Fernández-Villa, Antonio J Molina, Vicente Martín, Antoni Serrano-Blanco, Jordi Alonso, Mireia Espallargues

**Affiliations:** 1 Institute of Psychiatry, Psychology and Neuroscience King's College London London United Kingdom; 2 CIBER Epidemiología y Salud Pública Barcelona Spain; 3 Agency for Health Quality and Assessment of Catalonia Barcelona Spain; 4 Institute of Biomedicine University of Leon Leon Spain; 5 Health Services and Chronic Diseases Research Network Barcelona Spain; 6 Health Services and Policy Research Group University of Exeter Medical School Exeter United Kingdom; 7 Section of Personality, Assessment and Psychological Treatment Department of Clinical Psychology and Psychobiology University of Barcelona Barcelona Spain; 8 Parc Sanitari Sant Joan de Déu Barcelona Spain; 9 Health Services Research Group Hospital del Mar Medical Research Institute Barcelona Spain; 10 Department of Experimental and Health Sciences Pompeu Fabra University Barcelona Spain

**Keywords:** mobile technologies and social media, depression, adolescents, review

## Abstract

**Background:**

Despite the relevance of mobile technologies and social media (MTSM) for adolescents, their association with depressive disorders in this population remains unclear. While there are previous reviews that have identified the use of MTSM as a risk factor for developing depression, other reviews have indicated their possible preventive effect.

**Objective:**

The aim of this review was to synthesize the current evidence on the association between MTSM use and the development or prevention of depressive disorders in adolescents.

**Methods:**

An umbrella review was conducted using information published up to June 2019 from PubMed/MEDLINE, PsycINFO, Web of Science, and The Cochrane Library. Systematic reviews focusing on the adolescent population (up to 20 years old) and depression and its potential relationship with MTSM use were included. Screening of titles, abstracts, and full texts was performed. After selecting the reviews and given the heterogeneity of the outcome variables and exposures, a narrative synthesis of the results was carried out.

**Results:**

The search retrieved 338 documents, from which 7 systematic reviews (3 meta-analyses) were selected for data extraction. There were 11-70 studies and 5582-46,015 participants included in the 7 reviews. All reviews included quantitative research, and 2 reviews also included qualitative studies. A statistically significant association between social media and developing depressive symptoms was reported in 2 reviews, while 5 reviews reported mixed results.

**Conclusions:**

Excessive social comparison and personal involvement when using MTSM could be associated with the development of depressive symptomatology. Nevertheless, MTSM might promote social support and even become a point of assistance for people with depression. Due to the mixed results, prospective research could be valuable for providing stronger evidence.

## Introduction

Depression is one of the most frequently occurring mental diseases worldwide, generating significant disability, dependence, and expenditure for health systems [1-4]. As shown in previous literature [5-10], adolescence is a particularly relevant period for developing depressive disorders. It should be noted that during adolescence, depressive symptomatology may be broader than in adulthood, manifesting itself through irritability, aggression, avoidance, or other behaviors in addition to the typical depressive behaviors [11]. Furthermore, during this period, young people can be especially influenced by sociocontextual factors, such as the use of mobile technologies and social media (MTSM). However, the effect of the exposure to these technologies on the development of depressive disorders in this age group remains unclear.

The use of MTSM has greatly increased over recent years, particularly since the 1990s, and adolescents can now be considered “digital natives,” meaning they have been exposed to mobile devices and technologies like cellphones or tablets since birth [12-14]. This generalized exposure to social media implies a change in the way adolescents interact and communicate, naturally integrating the use of these technologies within their schemes of social perception [15,16]. Therefore, the use of MTSM could be particularly relevant, given the potential influence on adolescents’ health, specifically their mental health and the development or prevention of depression.

One of the main uses of MTSM among adolescents is communication and social interaction with their peer groups through various means, including instant messaging apps (eg, WhatsApp and social networks). A few that stand out for their use in this population are Instagram, Snapchat, Twitter, and Facebook [15,17]. Using MTSM could prove beneficial in the sense that they may promote creativity, increase presence and social participation, and provide adolescents with quick access to different types of information, including that related to promoting healthy behaviors and habits [12,13,18]. However, the use of MTSM could also be related to problems like addictive internet behavior, absenteeism and failure in school, deterioration of family relationships and friendships, and different physical and mental health problems (including self-inflicted bodily impairment, eating disorders, and depression) [12,13,19]. Furthermore, MTSM use may also promote behavior that is damaging to health including, among other things, autolytic behavior, suicide, violence, and specific harmful behaviors such as cyberbullying, grooming, or sexting that are derived from the use of these technologies. Despite the abundance of literature, including systematic reviews and meta-analyses, most of the existing evidence is based on cross-sectional studies or surveys. Pooling or synthesizing data and using the broadest possible approach (eg, an umbrella review) could be valuable in determining the current knowledge on whether the use of MTSM is the cause or consequence of depressive symptomatology.

Although there is a wide variety of advantages and disadvantages that the use of new technologies can present for young people, the influence that their use could have on developing depression is unclear. Therefore, the aim of this review was to synthesize the evidence available on the association (intensity and direction) between depression and the use of MTSM in adolescents.

## Methods

### Study Design and Information Sources

An umbrella review on the association between the use of MTSM and depression was conducted, reported in accordance with the Preferred Reporting Items for Systematic Reviews and Meta-Analyses criteria (PRISMA), and registered in PROSPERO. The following databases were used as sources of information: PubMed/MEDLINE, PsycINFO, Web of Science, and Cochrane Reviews. All documents included in these databases published up to June 2019 were considered.

A search filter (Multimedia Appendix 1) was specifically designed to achieve the study objectives, taking into account pathology, target population, exposure (social network OR social media OR mobile phone OR *phone), and the languages in which the search was performed. After carrying out a preliminary search and observing the number of systematic reviews and meta-analyses found as well as the differences between the studies, an additional filter for study design was included. The filter was designed for PubMed/MEDLINE and adapted for other databases. The search strategy was based on previous studies in other areas with the intention of maximizing the number of identified documents [20,21]. In addition, the references in the final selected studies were used to identify other systematic reviews and meta-analyses, and key authors were contacted.

### Inclusion and Exclusion Criteria

The PICO (Population, Intervention, Comparison, and Outcome) criteria were used to identify and include reviews in English that focused on the adolescent population (up to 20 years old), depression (in a broad sense, not specific diagnoses like major depressive disorder or dysthymia), and the possible relationship between depression and the use of MTSM.

Reviews that included studies with participants older than 20 years and studies that did not differentiate the effect by age group, if they included people older than 20 years, were excluded. Due to difficulties in extrapolating the results for the general adolescent population, studies on genetic or environmental factors and studies carried out in specific population groups, like those with specific characteristics or pathologies (eg, attention deficit hyperactivity disorder), were excluded. Finally, studies focusing on treatments administered through an electronic device or the internet as well as opinion articles and proposals with theoretical or conceptual frameworks that were not based on a systematic literature review or meta-analysis were also excluded.

### Review Process 

A review of titles, abstracts, and full texts was carried out independently by two expert reviewers (JAT and XG), and discrepancies were resolved by a third researcher (EP) with expertise in conducting systematic reviews. After study selection, a synthesis of the evidence obtained from the 7 selected reviews was carried out. The quality of each review was considered by taking into account the quality of the studies evaluated and the tools used to assess the studies. Owing to heterogeneity in the characteristics of the studies and in the presentation of outcome variables and exposures, a meta-analysis of the results was not possible; therefore, a narrative synthesis of the results was carried out. Information from the included reviews was extracted and summarized in 2 tables of evidence [22].

## Results

**Figure 1:**
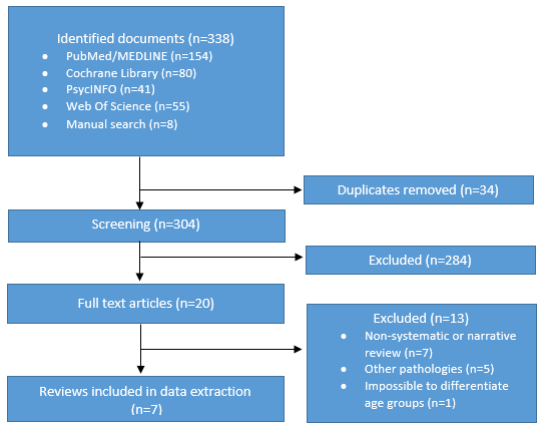
Flow diagram of the review process.

The search retrieved 338 articles (154 from PubMed, 80 from the Cochrane Library, 41 from PsycINFO, 55 from Web of Science, and 8 from a manual search). After removing 34 duplicates, a total of 304 studies were deemed potentially eligible. The full text of 20 documents was reviewed, and 13 articles were excluded (7 non-systematic or narrative reviews, 5 documents based on other pathologies, and 1 for the inability to differentiate between results reported for adults versus adolescents). Finally, 7 systematic reviews were selected for data extraction (Figure 1) [21,23-30].

Table 1 shows the characteristics of the included systematic reviews, all of which were published between 2014 and 2019. In these reviews, PsycINFO, Medline, and CINAHL databases were searched most frequently. Two reviews explored dissertations and thesis databases [25,31]. Most reviews assessed the relationship between depression and use of social networks in general [23,25,26,28] or problematic Facebook use in particular [31]. One study by Wu et al [29] reviewed the association between internet use in general and depression. Wellbeing, anxiety, and loneliness were also assessed in 2 reviews [26,29]. There were 11-70 studies and 5582-46,015 participants included in the reviews. Most studies included in the reviews were quantitative and used cross-sectional and survey-based data. While 2 reviews used specific criteria developed by the authors to assess the quality of studies [29,31], 4 used validated assessment tools [21,23,26,28], and 1 did not specify the tool [25]. In addition, 2 meta-analyses were included [25,31].

Table 2 shows the results of the included reviews. Four studies were undertaken specifically with adolescents (age range 10-21 years) [21,23,28,29]. Seabrook et al [26] also included adults in their review (2 studies with adults and 18 studies with the general population), and Marino et al [31] reported a mean age range of 16.5-32.4 years. While 2 reviews reported a positive association between depressive symptoms and social media use (overall random effects pooled estimate: *r*=0.13, 95% CI 0.05-0.2) [23] and problematic Facebook use (*r*=0.34, 95% CI 0.28-0.39) [31], the other 5 reviews reported mixed associations between social media use and depression. Keles et al [28] reported a positive association for the relationships between time spent on social media and depression and between social media addiction and depression. Two reviews reported a gender influence with mixed effects [23]. McCrae et al [23] found that 4 studies reported girls having more depressive symptoms related to social media use and 2 studies showed that boys were more likely to show depressive symptoms. The rest of the studies included in their review did not show a gender effect. In the review by Keles et al [28], one study found that social media might have negative effects in girls but could be considered a positive leisure activity for boys, and 2 studies did not show gender effects. In addition to mixed results for the associations between social media use and wellbeing, associations with anxiety and loneliness were also found [21,26,29].

**Table 1:** Characteristics of the included reviews.

Author (year)	Objective of the review	Databases searched	Number of studies included	Number of participants	Quality assessment of studies included	Methodology
Best et al (2014) [21]	To assess the impact of social media use on mental wellbeing in young people	ASSIA^a^, Communication abstracts, CINAHL, ERIC^b^, Medline (Ovid), PsycINFO (Ovid), SCOPUS, SSCI^c^	43	NS^d^	Specific criteria developed by the authors of the review	32 quantitative, 9 qualitative, 2 mixed methods or others
Wu et al (2016) [29]	To examine the association between internet use, social connection, and levels of depression, anxiety, and loneliness	CINAHL, ERIC, Psychology and Behavioral Series Collection, Science and Technology Collection, EBSCO social sciences database	12	5582	Specific criteria developed by the authors of the review	9 quantitative (all cross-sectional), 1 mixed methods, and 2 qualitative
Seabrook et al (2016) [26]	To examine the relationship between the use of social networks and depression and anxiety as well as links with wellbeing and potential mediators and moderators of these relationships	PsycINFO, MEDLINE (Ovid), Scopus, IEEE Xplore, CINAHL, Education Resources Information Center, SSCI, Communication and Mass Media Complete	70	46,015	Adaptation of the Cochrane bias tool	NS
McCrae et al (2017) [23]	To examine the association between social media (websites used primarily for social interaction) and depression or depressive symptoms	Medline, PsycINFO, EMBASE	11	12,646	Robins-I^e^, Cochrane Collaboration Methods Group Tool to assess risk of bias in cohort studies	Quantitative (7 cross-sectional, 4 longitudinal)
Marino et al (2018) [31]	To examine the association between Facebook use (problematic, abusive, overuse, compulsive) and psychological disorders in adolescents and young adults	PsycINFO, PubMed, Scopus, ResearchGate, Google Scholar, Dissertation Abstracts International, Pro-Quest Dissertations and Theses Open, Open Access Theses and Dissertations	23	13,929	Specific criteria developed by the authors of the review	Quantitative
Keles et al (2019) [28]	To examine the influence of using social networks on depression in adolescents	PsycINFO, Medline, EMBASE, CINAHL, SSCI	13	21,231	NIH^f^	Quantitative (12 cross-sectional, 1 longitudinal)
Yoon et al (2019) [25]	To examine the relationship between the use of social networking sites and depression	PsycINFO, PubMed, ProQuest Dissertations & Theses Global	55	22,099	NS	Quantitative

^a^ASSIA: Applied Social Sciences Index and Abstracts.

^b^ERIC: Education Resources Information Center.

^c^SSCI: Social Sciences Citation Index.

^d^NS: not specified.

^e^Risk of bias tool to assess nonrandomized studies of interventions.

^f^NIH: National Institutes of Health Quality Assessment Tool for Observational Cohort and Cross-Sectional Studies.

**Table 2:** Results of the included reviews.

Author (year)	Sample (number of studies) or age (years)	Use of MTSM^a^	Association(s)	Gender effect	Other associations
Best et al (2014) [21]	Adolescents (age range not specified)	Communication and social interaction	Mixed results in the association of social media technologies and depression	Does not distinguish nor consider this factor	Mixed results on self-esteem, social support, loneliness, and cyberbullying
Wu et al (2016) [29]	10-21	Use of internet and related technologies	1 of 5 studies found that social media technology use can lead to depressive feelings; 4 of 5 studies did not find an association.	Takes into account the population of the studies (10 mixed gender, 2 only boys), but not in terms of the results	Mixed results on social connectivity, anxiety, and loneliness
Seabrook et al (2016) [26]	Adolescents (8), young adults (40), general population (18), adults (2), clinical depression (1), others (1)	Use of social networks	Mixed results: positive interactions, social support, and connectivity in social networks related with lower levels of depression; negative interactions and social comparison related with higher levels of depression	Not considered as a variable in the included studies but considered in the discussion of the results	Mixed results for anxiety and wellbeing
McCrae et al (2017) [23]	10-17 (one study included “high school students” but did not specify age range)	Use of social media	Small but statistically significant overall correlation between social media use and depressive symptoms	4 studies found that girls had more depressive symptoms related to social media use; 2 studies showed that boys were more likely to show depressive symptoms; the rest showed no gender differences	NS^b^
Marino et al (2018) [31]	Mean 21.9 (SD 3.97); 16.5-32.4 (mean age range)	Problematic Facebook use	Association between problematic Facebook use and depression	Proportion of girls (60.7%) did not moderate the effect	Correlation between problematic Facebook use and psychological distress was greater in samples with a higher mean age.
Keles et al (2019) [28]	13-18	Time spent, activity (quality and quantity of user’s engagement and interaction with social media sets and other users), investment (time spent on social media), addiction (state of being dependent on social media)	Time spent: 1 study showed association, 1 did not, 2 did not find association; activity: 2 studies showed positive association, and 1 did not; investment: 3 studies showed association; addiction: 3 studies showed positive association	4 studies measured the effect of gender between social media–related variables and mental health outcomes. 2 studies did not find effects on gender, while 1 found that social media might have negative effects in girls and can be considered a positive leisure activity for boys. Facebook had a negative impact on both genders.	There was a relationship between age, heavy social media use, and negatively internalizing symptoms. Younger adolescents were more likely to experience internalizing symptoms (being anxious, depressed, withdrawn). Most studies highlighted the fact that the relationships observed were too complex for straightforward statements and mediating and moderating factors should be taken into account.
Yoon et al (2019) [25]	17.83-24.76 (mean age range)	Use of SNS^c^: time spent and SNS checking; social comparison and “upward” social comparison	Positive statistically significant difference between depression and time spent on its use, frequency of use, social comparison, and “upwards” comparison	No difference	NS

^a^MTSM: mobile technologies and social media.

^b^NS: not specified.

^c^SNS: social networking sites.

## Discussion

The results from the included reviews suggest that social comparison and excessive personal involvement by adolescents when using MTSM could be related to the development of depressive symptoms. However, the use of MTSM when properly adapted could also promote healthy behaviors, improve social support, and even become a point of access of information and help for adolescents at risk of depression.

Both mobile technologies and social media are important aspects of how we interact today and have transformed the way in which the generations adopting MTSM and digital natives communicate [18,32]. The use of MTSM presents great opportunities in terms of creativity and ways of learning but can also entail certain risks such as isolation and restricted social interaction. Despite this, studying the possible effects on health, specifically on depression, of adolescents using MTSM is a relatively recent phenomenon. As such, it should be noted that all reviews included in this study were published in the last 5 years.

The evidence from different studies published until now, and particularly since 2017, suggests a positive and significant association between some aspects of social media use and the presence of depressive symptoms among adolescents [23-25]. Two relevant factors that increased the magnitude of this association were the problematic use of social networks and excessive social comparison [23-25]. There is less relevant evidence pointing to other factors related to the undesirable effects of social networks, like a higher level of personal involvement on the networks, defined as the degree of exposure and personal information that adolescents publish on networks or the exposure to content that promotes depressive-like behaviors [27,28]. Finally, it is worth mentioning that a high volume of studies indicating associations between the use of social networks and other undesirable effects like anxiety, harassment, or internet or smartphone addiction was identified [21,26,28-30]. Regarding internet addiction, the total usage time, frequency of consultation, and other variables related to excess use, both in frequency and time, may be more relevant than the variables found in this study, which focus specifically on depressive symptomatology.

It should be noted that the impact of the identified factors, particularly of social comparison, on the development of depression might be affected by the level of the welfare and wealth of the family [7-12]. Accordingly, those who are from families with lower socioeconomic status might have a high risk of developing depression when exposed to more wealthy people. In addition, these factors might be particularly related to the development of some specific depressive symptoms (eg, sleep problems or diminished ability to think or concentrate). Further longitudinal research focused on specific factors, like family environment, and accounting for specific depressive symptoms might be valuable in preventing the potential development of depression in MTSM users.

Emphasizing the fact that social networks do not necessarily imply a negative impact on young people’s moods, other studies have described the desirable effects that social media use might have [12,33,34]. In this sense and in line with the results of these studies, the evidence found in this study suggests that social networks can promote social support and even become points of access to information and help for people with depressive disorders [26,27]. As suggested, the use of MTSM under adult supervision might be related to promoting healthy use of MTSM, as well as preventing possible negative consequences that arise like depressive symptomatology [35]. In addition, the use of new technologies could facilitate young people’s connection with multiple social circles, reducing their perception of loneliness or isolation [29].

Some studies identified differences between boys and girls in the impact that social networks have on developing depressive symptoms. Previous research proposed [30] that the prevalence of intensive use of mobile technologies might be greater in women than in men. Furthermore, the use of mobile technologies could be mainly for relational purposes among teenage women and instrumental or for leisure among teenage men, making women more likely to be exposed to the effects of social networks [23,24,28]. Although the meta-analysis by McCrae et al [23] did not determine a theoretical basis for the potential differences, there are some studies included within the analysis and 1 study included in the systematic review by Keles et al [28] that show a greater correlation between social comparison and depression in women. This might allow us to hypothesize that focusing preventive measures on social comparison in adolescent women and on leisure platforms, like gaming platforms, in adolescent men could be effective in preventing the undesirable effects of social networks and mobile technology use among adolescents. Further research aimed at proving this hypothesis could be valuable.

Several limitations of the current study deserve discussion. First is the lack of longitudinal or experimental evidence in relation to the use of social networks and mobile technologies and their impact on depressive symptomatology. In this sense, most of the studies included in the literature reviewed were cross-sectional and survey-based, precluding the establishment of causal relationships between variables. As such, it is difficult to determine whether the use of social networks and mobile technologies is the cause or consequence of depressive symptomatology, and further longitudinal studies to test these hypotheses could be valuable. We should also mention the possible heterogeneity of health problems and of the patterns made or activities observed in the studies when using MTSM. While some were focused on clinical depression diagnosed by a professional, others were focused on less valid depression criteria, which could limit the comparability of the reviews included. Furthermore, some of the reviews included internet addicts. Despite this, the broad aim of this review was to determine the relationship between depressive disorders and the use of MTSM, which we consider completed through the studies included in this article, independent of the depression metrics and specific populations used in the selected reviews.

Another limitation is the lack of solid evidence or a conceptual framework on the specific behaviors, like online gaming or uploading photos to social networks, that could be related to depressive symptomatology. This lack of evidence may be due to the relative novelty of the social network phenomenon and the shortage of valid, reliable health information pertaining to it. However, certain behaviors that could be related to the development of depression as a protective factor were identified, like searching for help or preventive information. Another limitation is that only reviews in English were included, possibly omitting scientific literature written in other languages. Finally, we should mention the limitation of having actively excluded studies on cyberbullying, addiction to new technologies, or other symptoms and harmful behaviors that could be part of or related to a depressive disease. Given their importance and the abundance of evidence on these phenomena, these behaviors deserve to be treated as separate entities, and, as previous research suggests, specific reviews should be performed on these behaviors [21,26].

In conclusion, our study shows that, during adolescence, the use of MTSM and particularly excessive social comparison and personal involvement when using it could be associated with developing depressive symptomatology. Nevertheless, the adaptive use of MTSM could also help prevent the development of depression, promote social support, and even become a point of information access and help for people with depressive disorders or symptoms. Other variables, like time spent on the internet and social networks, the frequency of consultation, and factors related to excess use, both in frequency and in time, may be more relevant in developing other problems like internet addiction. Due to the heterogeneity in methodology and the contradictory findings from the reviews included in this umbrella review, prospective research, especially longitudinal cohort studies and randomized controlled trials, could be valuable in providing stronger evidence on these relationships.

## Appendix

Multimedia Appendix 1 PubMed/MEDLINE filter.

## Abbreviations

ASSIA: Applied Social Sciences Index and Abstracts
ERIC: Education Resources Information Center
MTSM: mobile technologies and social media
NIH: National Institutes of Health Quality Assessment Tool for Observational Cohort and Cross-Sectional Studies
NS: not specified
PICO: Population, Intervention, Comparison and Outcome
PRISMA: Preferred Reporting Items for Systematic Reviews and Meta-Analyses criteria
SSCI: Social Sciences Citation Index
