# Supplementary material for: Relationship Between Depression and the Use of Mobile Technologies and Social Media Among Adolescents: Umbrella Review
Source: J Med Internet Res. 2020 Aug 26;22(8):e16388. doi: 10.2196/16388 (PMC7481866; doi:10.2196/16388)
Supplement: Multimedia Appendix 1 [file jmir_v22i8e16388_app1.docx]

## Multimedia Appendix 1

PubMed / MEDLINE filter

#1 Pathology

(Depres*)

#2 Population

(Adolesc* OR teen*)

#3 Exposure

(Social network OR social media OR mobile phone OR *phone)

#4 Type of study

(Systematic Review[pt] OR meta-analysis[pt] OR review [pt] OR Systematic Review [mesh] OR meta-analysis [mesh] OR systematic review OR meta-analysis OR review)

#5 Language

(English)

#6 #1 AND #2 AND #3 AND #4 AND #5
